# Growing up in Ancient Sardinia: Infant-toddler dietary changes revealed by the novel use of hydrogen isotopes (δ^2^H)

**DOI:** 10.1371/journal.pone.0235080

**Published:** 2020-07-08

**Authors:** Saskia E. Ryan, Linda M. Reynard, Elisa Pompianu, Peter van Dommelen, Clizia Murgia, M. Eulàlia Subirà, Noreen Tuross

**Affiliations:** 1 Department of Human Evolutionary Biology, Harvard University, Boston, Massachusetts, Unitied States of America; 2 Independent researcher, Marrubiu, Italy; 3 Joukowsky Institute for Archaeology and the Ancient World, Brown University, Providence, Rhode Island, United States of America; 4 Departament de Biologia Animal, Unitat d’Antropologia Biològica, Biologia Vegetal i Ecologia, Universitat Autònoma de Barcelona, Barcelona, Spain; 5 Grup de Recerca en Antropologia Biològica, Unitat d’Antropologia Biològica, Universitat Autònoma de Barcelona, Barcelona, Spain; Senckenberg Gesellschaft fur Naturforschung, GERMANY

## Abstract

Detailed information about the lives and deaths of children in antiquity is often in short supply. Childhood dietary histories are, however, recorded and maintained in the teeth of both juveniles and adults. Primary tooth dentinal collagen does not turn over, preserving a sequential record of dietary changes. The use of nitrogen (δ^15^N) and carbon (δ^13^C) isotope values of incrementally sampled dentin are used in the study of breastfeeding practices but evidence for the addition of weaning foods, both in terms of mode and, particularly, duration, has remained analytically inaccessible to date. Here, we demonstrate how the novel use hydrogen isotope (δ^2^H) values of sequentially micro-sampled dentin collagen, measured from individuals excavated from a Punic cemetery, in Sardinia, Italy, can serve as a proxy for weaning food type and duration in ancient childhood diet. The weaning rate and age, based on the decline in δ^15^N and δ^13^C values of permanent first molars and the concomitant increase in δ^2^H, appears to be broadly similar among six individuals. Hydrogen isotopes vary systematically from a low value soon after birth, rising through early childhood. The early post-birth values can be explained by the influence of ^2^H-depleted lipids from mother’s breastmilk and the later δ^2^H rise is consistent with, among other things, a substantial portion of boiled foodstuffs, such as the higher δ^2^H values observed in porridge. Overall δ^2^H in dentin shows great promise to elucidate infant and childhood feeding practices, and especially the introduction of supplementary foods during the weaning process.

## Introduction

The interaction of infectious disease and malnutrition have shaped the trajectory of human evolution. Nowhere are these effects more pronounced than in infant survival. Today, the global under-5 mortality rate is 3.9% [1], but the extent of infant mortality was much greater in the past. Even as late as the mid 1800s in Italy, our greater study location, approximately 20% of infants succumbed to a number of diseases, including gastrointestinal disorders, pneumonia and bronchitis, by the age of one [2]. The introduction of weaning foods often exacerbates the exposure to disease-causing microorganisms and extends the high infant mortality rates into toddlerhood. Weaning-related deaths existed in the past and persist as a source of childhood mortality to this day. So, while the death rates of infants up to one year of age are estimated at around 25% for much of human history [3], the second year of life comes with substantial risk as infants are weaned from human milk [4]. Although some studies have documented the complex nature of infant feeding (e.g.[5]), our knowledge of ancient infant feeding and nutrition remains limited. When textual sources are lacking, infants and young children become even less visible. Archaeological studies underrepresent many aspects of infancy and childhood [6] due in part to the relative scarcity and preservation of skeletal remains. In addition, infants and children who are part of the archaeological record do not necessarily reflect the healthy child population that survived to adulthood [7–11].

Bioarchaeology has contributed to our knowledge of infant and child lifeways [12,13] and the incorporation of stable isotopic values in the study of breastfeeding and weaning has become standard [8,10,14], albeit not without challenges. The empirical observation that infant bone collagen has elevated amounts of ^15^N as compared to adults was first reported in 1989 [15], and followed by a study of an archaic population from Florida (USA) that exhibited elevated ^15^N in bone collagen of children vs the adults [16]. As an isotopic medium to interpret infant feeding patterns, bone is often hampered by population sample size [8], the time averaging of protein synthesis, and the unknown health status of individuals: all of these issues cause fine grained determinations of breastfeeding duration from bone to be questionable.

Many of the difficulties associated with the interpretation of bone isotopes can be overcome by the use of serial sections of dentin from both children and adults, as primary dentin maintains the dietary inputs of infancy in the form of isotopic trends [14,17–24]. Most archaeological studies of breastfeeding and weaning practices have been entirely focused on the use of nitrogen and carbon isotopes; less frequently, oxygen isotopes, strontium and calcium concentrations, and calcium isotope ratios have been used (see [14] for a review). Here, we present a new approach—the hydrogen isotope composition of tooth dentin—to independently document the duration of breastfeeding and, for the first time, provide an isotopic record of the potential range of foods used in the weaning process from individuals excavated from contexts dating from the 4^th^ to early 2^nd^ century BCE, from a Punic cemetery in Villamar [25–27], on the island of Sardinia, Italy.

Hydrogen isotopes are powerful biogeochemical tracers; the hydrogen (and oxygen) isotope composition of surface and groundwater vary geographically, typically reflecting the isotope ratio of precipitation [28]. This variation in geographically distributed isotopes can be subsequently reflected in human and faunal tissues [29–32] along with the contribution of δ^2^H and δ^18^O from food [33,34]. Water δ^2^H in cow’s milk is somewhat higher on average compared with co-occurring drinking water [35–37], and milk lipids have been found to be uniformly lower in δ^2^H values compared to either liquid source [38,39]. This finding highlights the role of the isotopic composition in different milk components for the interpretation of infant dentin collagen δ^2^H values. Previous reports suggest that δ^2^H in bone collagen is related to trophic level [40–45]; however, this is not universally observed, and because these reports refer to cross species comparisons, the relevance to very young human infants is unknown.

Another potential controlling factor of dentin δ^2^H values is food preparation practice. Food and water sources can be isotopically fractionated through boiling, fermentation, distillation and cooking, though very few studies have measured the δ^2^H values of cooked food [46,47]. The introduction of heavily cooked foods, for example, cereal gruel, which has historically been used as a transitional food during the weaning period, could influence dentin δ^2^H values. Barley and wheat have played an instrumental role in the subsistence of Sardinian residents historically [48]; botanical remains show a continuous record of barley cultivation from at least the Nuragic Bronze Age to the Middle Ages and beyond (1500 B.C.—A.D. 1100) [49]. Naked wheat (*Triticum aestivum-durum*) was also found to be in abundance in Punic contexts elsewhere in Sardinia [50], but it is thought that wheat was exported while barley was the cereal that was consumed by inhabitants [51]. In addition to the sequential dentin collagen samples, this study presents δ^2^H values of both human breastmilk and porridge, a canonical weaning food, to assess their potential effect on dentin isotope composition.

## Materials and methods

### Site and individuals

**Fig 1:**
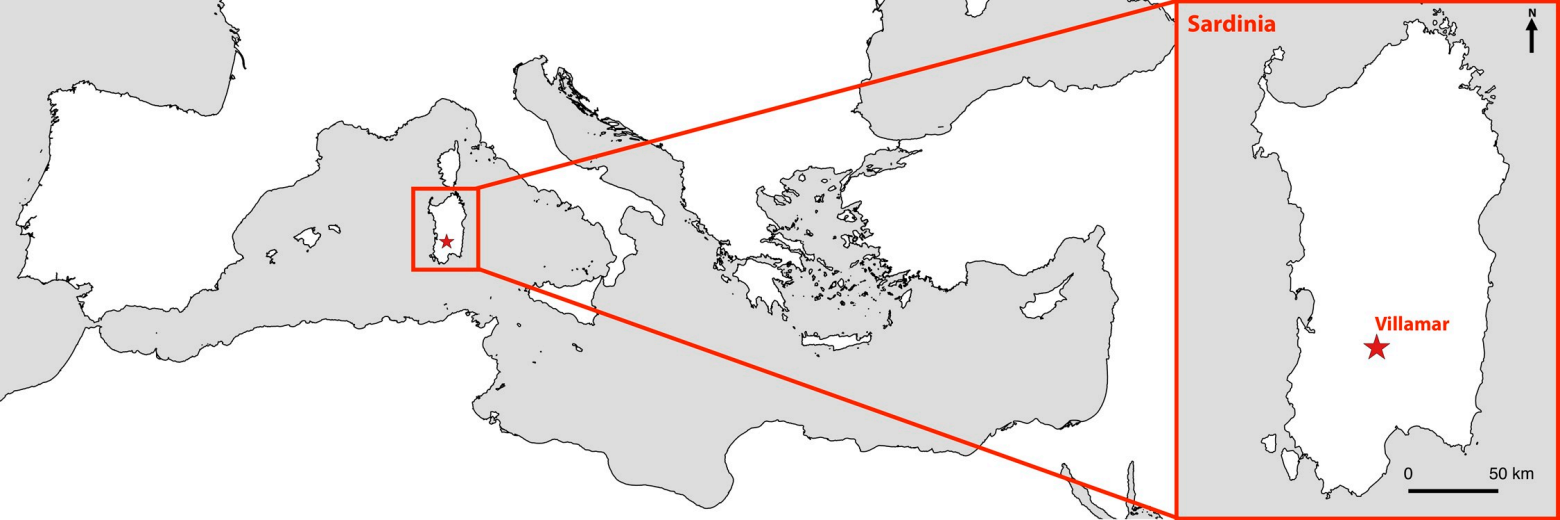
Villamar in Sardinia, Italy, where the cemetery from which the individuals were excavated is located. The freely available shapefile from the Ancient World Mapping Centre (AWMC) [52] was used as the basemap and modified with QGIS software [53].

We studied six human individuals and three caprids from the site of Villamar in central Sardinia (Fig 1)(S1 Table for details). Authorization for the use and exportation of the Sardinian specimens was granted by the Soprintendenza Archeologia Belle Arti e Paesaggio per la città metropolitana di Cagliari e le province di Oristano e sud Sardegna, with prot. 15667 of 28 July 2017. Official: Chiara Pilo. All necessary permits were obtained for the described study, which complied with all relevant regulations. The site is a Punic cemetery (4^th^ to early 2^nd^ century BCE), where 25 of approximately 50 known tombs have been excavated since 2013 [25–27](S2 Table for radiocarbon dates). A total of 299 isotopic values (δ^2^H of dentin samples = 86 and bone collagen = 9; δ^15^N and δ^13^C of dentin samples = 93 and bone collagen = 9) were produced from these 6 humans and 3 caprids. The cemetery occupies a rocky outcrop of marl and sandstone into which the graves were cut. The prevalent burial ritual is inhumation, with most of the deceased having been buried in either chamber tombs or rock-cut niches, accessible via a vertical and roughly rectangular shaft (See S1 Appendix for further site information).

### Incremental dentin sampling

Reconstructing breastfeeding and weaning practices (BWP as defined by Tsutaya & Yoneda 2014[14]) is made possible by micro-sampling dentin and/or enamel [22,54–56]. For this study, the tissue of choice is dentin collagen from the first permanent molar due to its relatively straightforward developmental pattern, the size of the sample required for measurement and the ability to measure hydrogen isotopes in this tissue. Dentin formation commences prior to enamel development and starts at the crown, extending progressively towards the root [57]. Matching the biological age to the incremental sections of dentin cannot be done with complete accuracy as the dentin grows in stacked cones [58] and serial sections are taken horizontally [14], thus cutting across overlapping dentin layers which have been deposited at different times. Therefore the values measured from a given section represent a moving average of the inherent isotope mixing [55]. We have taken the approach here of assigning estimated ages based on the midpoint of the section, and based on the AlQahtani Atlas (2010) [59]. The age at which M1 dentin formation begins is approximately 4.5 ± 3 months and the age at apex closure is approximately 9.5 ± 0.5 years.

The method of sectioning the dentin was adapted from ‘Method 2’ used by Beaumont et al., 2013 [60], which in turn was based on the work of Kirsanow et al., (2008) [55]. Using a rotary blade in a dental hand piece, each tooth was cut in half to isolate one complete root and adjoining section of the crown. Samples were photographed, sonicated in deionized (dI) water, and immersed in 0.5 M EDTA (pH 7.3) which was replaced every 1–2 days until complete demineralization. Dentin was rinsed 15 times in dI water and sequentially cut with a scalpel into thin parallel sections from the coronal dentin horn to the root cervix in 1 mm increments, perpendicular to the central axis of the root. Demineralized dentin sections were freeze-dried. The sampling procedure generated between 12 and 22 serial sections per tooth for a total n = 105.

### Bone sample preparation

Bone was sampled in order to gauge later-life isotope values [61–63]. Bone was demineralized in 0.5 M EDTA (pH 7.3) and washed 15 times with deionized water [64]. All of the bone collagen was well-preserved based on the criteria of atomic C/N ratios [65,66].

### Cooking experiment

Barley (*Hordeum vulgare*) was cooked in water at a temperature of 100°C for 135 minutes. The cooking water, partially-soluble barley fraction and insoluble barley fraction (grains) were sampled at 15 minute intervals throughout the cooking period and their respective hydrogen isotope values were measured.

### Breastmilk (BM) and urine separation

One mother and infant pair was recruited from the general region near Cambridge, Massachusetts, USA, and the mother gave written permission for sample collection. The University Area Institutional Review Board of Harvard University granted permission for human tissue from the mother and infant to be used in this study (IRB14-3463). The mother’s diet was omnivorous and unconstrained. Samples were collected periodically over a ~5 month period, beginning approximately two weeks after the infant’s birth. Expressed milk (n = 5) and urine (n = 7) samples were collected from the mother, and feces were collected from her infant baby (n = 5). The samples were frozen immediately after collection. One millilitre of defrosted milk was centrifuged at 3500 rpm for 30 min at room temperature to separate the lipid-protein mixture from the less dense water-carbohydrate components (BM water). The solid lipid-protein fraction was refrigerated and the lipids (BM lipid-rich) separated from the remaining mixture (BM lipid-depleted). After both fractions were sampled individually, they were homogenised and re-measured (BM solids). Urine samples were centrifuged at 3500 rpm for 30 min at room temperature and the supernatant (urine water) was collected for analysis. A fraction of the urine was also freeze-dried to facilitate transfer to silver capsules for analysis (urine solid). Breastmilk fluid and urine were filtered through 0.45 μm syringe filters prior to isotopic measurements.

### Isotope measurement

Solid samples—dentin, collagen, barley, various milk non-water fractions, urine solids, and feces—were measured by continuous-flow mass spectrometry at Harvard University on a Thermo-Finnegan Delta Plus XP. Dentin and bone collagen δ^15^N and δ^13^C values were obtained via a Costech Elemental Analyser, using USGS 40 and USGS 41 glutamic acid as standards, and data are reported relative to AIR for δ^15^N and VPDB for δ^13^C. The δ^2^H values of solid materials were obtained using a Thermo Thermal-Conversion Elemental Analyser (TC/EA), previously described in detail [64]. The δ^2^H values of bone collagen, infant feces, breastmilk solids and urine solids were analysed using a chromium (Cr)-packed reactor, as described in Reynard et al., 2019 [67]. A subset of dentin samples was analysed at the United States Geological Survey, also using a Cr-packed reactor (see S3 Table for further information). Tooth dentin δ^2^H measurements were obtained with a glassy carbon furnace packing and were converted to equivalent Cr-reactor-run values using the conversion factor that was also presented by Reynard et al., 2019 [67]. Samples were analysed in duplicate where possible and all δ^2^H data are normalised to the VSMOW-SLAP scale, using VSMOW and SLAP enclosed in sealed silver tubes as standards. The average standard deviation of the duplicate samples of bone collagen δ^2^H was ± 3 ‰ and 2 ‰ for tooth dentin (S3 and S4 Tables). The long-term keratin standard value is -89 ± 4 ‰ for Cr-δ^2^H (n = 63). Breastmilk water and urine water were analysed on a Los Gatos Research T-LWIA-45-EP liquid-water analyser for δ^2^H and δ^18^O and are reported relative to SMOW.

## Results and discussion

### Isotopic changes in dentin collagen during childhood

**Fig 2:**
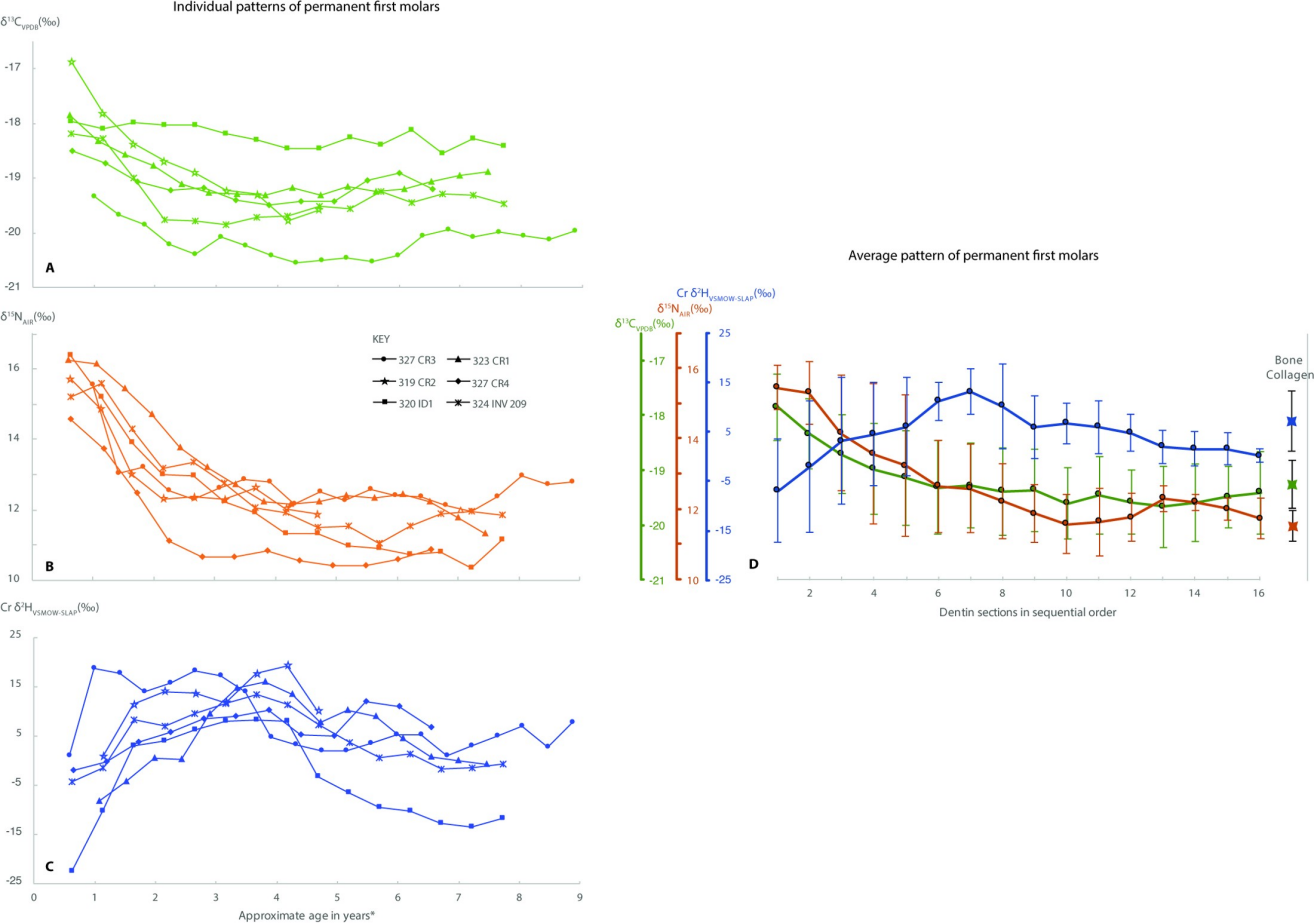
The left panels show the separate carbon (A), nitrogen (B) and hydrogen (C) isotope values from each individual (n = 6) from the incremental first permanent molar dentin sections. A range of ~1–3 ‰ in δ^*13*^C values, (A), ~4–5 ‰ in δ^*15*^N (B) and ~14–29 ‰ in δ^*2*^H values (C) is noted across the total length of tooth dentin. The right panel (D) shows the average values of the permanent first molars from 6 different individuals. For the purpose of visual representation, each data point from a 1 mm section is plotted in sequential order. *Approximate age in years is based on the commencement and cessation ages of tooth dentin determined by AlQahtani et al., (2010) [59], assuming a constant growth rate of dentin, factoring in the length of the tooth. The average length of the M1s sampled is used in the case of ‘319 CR2’ and ‘320 ID1’ whose dentin was not fully mineralised.

Each of the six individuals have strikingly similar trends in δ^13^C and δ^15^N values in their M1 throughout childhood, suggesting similar weaning practices in this population (Fig 2A and 2B, respectively). We note that both δ^13^C and δ^15^N values progressively fall during the formation of the M1 dentin indicating that less and less breastmilk was part of the toddler diet with increasing age. The δ^13^C and δ^15^N values gradually plateau in toddlerhood, reaching values close to those of the bone collagen (representing the years before death–adulthood in most cases here) (Fig 2D). Two individuals (US 320 ID1 and US 319 CR2), who died before reaching adolescence, marked by the cessation of root growth, do not have notably different δ^15^N patterns compared with those who survived into adulthood, suggesting no identifiable N isotope bias due to childhood mortality.

Hydrogen isotope patterns are dramatically distinct from the corresponding δ^15^N and δ^13^C weaning curves with an inverse pattern seen in the earliest years of dentin deposition (Fig 2D). From the earliest post-birth time segment in the M1 dentin, δ^2^H values rise rapidly until they peak and begin to decrease again, wavering through later childhood, with values in the latter sections closely corresponding to those of the average bone collagen δ^2^H value (Fig 2C and 2D). As the isotopic patterns of permanent M1 dentin collagen do not reflect dietary input right at birth, both the high δ^15^N and δ^13^C values may have already declined from a maximum value. The permanent M1 dentin collagen does, however, capture the total trajectory of non-breastmilk dietary introduction to the infant/toddler.

### Dietary sources contributing to dentin collagen isotope composition

#### Nitrogen

The nitrogen source in the diet of monotonously breastfed infants is milk supplied from the mother. This milk source reflects the diet of the mother and in an agricultural community such as Villamar, the adults of the community were likely omnivores. The δ^15^N of bone collagen from Villamar averaged 11.5 ± 0.5 ‰ (n = 6), consistent with an adult diet that contained meat products. For two reasons, we argue that the likely source of nitrogen in weaning foods were likely plant based. First, the average δ^15^N of caprid bone collagen (n = 3), is 10.5 ± 0.9 ‰, only 1 ‰ lower than the co-occurring adults at Villamar, making caprid milk or meat an unlikely major dietary component for human weanlings. Second, the total average decline from in M1 dentin δ^15^N collagen was 4.3 ± 0.5 ‰ (average ± SD total range, n = 6), consistent with a mixed weaning diet that contained largely plant derived products.

#### Carbon

The δ^13^C values of dentin mirror those of δ^15^N (Fig 2D), declining by on average 1.5 ± 0.8 ‰ (n = 6) over the time represented. The drop in δ^13^C is consistent with an enriched animal (mother) input followed by the introduction of C_3_ plant-based weaning foods with lower δ^13^C values.

#### Hydrogen

**Fig 3:**
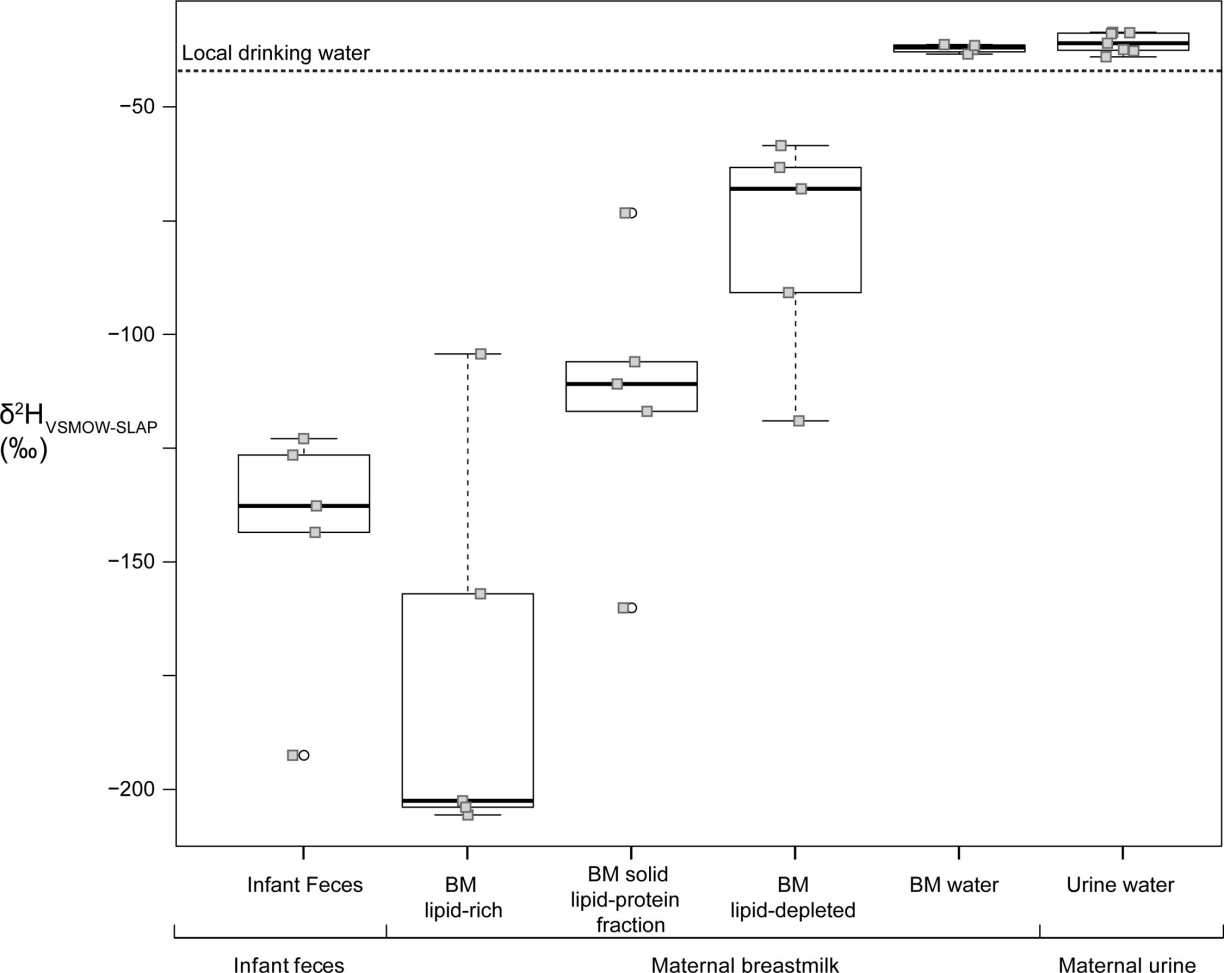
Hydrogen isotope values of infant feces, and maternal breastmilk fractions and urine water. See S5 Table for data. Open circles are outliers. Solid samples were analysed using a Cr-packed reactor.

Far less is known about the hydrogen isotope composition of potential weaning foods, and for that reason we undertook two types of analyses. From a mother-baby pair, we observe human breastmilk water δ^2^H values to be slightly higher than that of local drinking water values (Δ_average of breastmilk water- drinking water_ = 4.8 ± 1.5 ‰), in agreement with observed ^2^H enrichment in cow's milk water over drinking water [38,68]. In addition, as previously reported [69], human breastmilk and urine water were isotopically indistinguishable in δ^2^H. The non-water fractions are all significantly ^2^H depleted vs. both the breastmilk water and the local drinking water (Fig 3), in agreement with previously reported data from cows [38].

The hydrogen isotope composition of the infant/toddler dentin is depleted in ^2^H in early infancy (Δ_average of dentin horn section-bone collagen_ = -15.0 ± 5.8 ‰ of earliest M1 dentin vs. bone collagen) and steadily rises over the period in which the nitrogen isotope patterns would suggest weaning is taking place. It is likely that lipid-rich human breastmilk is a sole dietary source during the early stages of infancy and that this monotonous food source significantly contributes to the early depletion in ^2^H.

The simultaneous high δ^15^N, δ^13^C and low δ^2^H in the early-formed dentin segments demonstrates that δ^2^H values in dentin are not elevated during exclusive breastfeeding. The dentin δ^2^H values are not “trophic” in the sense used in some hydrogen isotopic literature (referring to herbivores vs. omnivores vs. carnivores) [40–45], but rather reflect early milk inputs.

The rise in δ^2^H values in the M1 dentin is inconsistent with a diet dominated by animal products, and in agreement with the nitrogen isotopic data which suggest that a substitute animal milk source was not a major contribution to the infant diet during the weaning period. There are many other possible foods that could have been used as a substitute food source for weaning infants of Villamar, including likely soft sources such as fruit which often have elevated δ^2^H values relative to precipitation [70–72]. We chose to test whether cereal gruel/porridge hydrogen isotope values were consistent with the observed increase in the infant dentine collagen.

**Fig 4:**
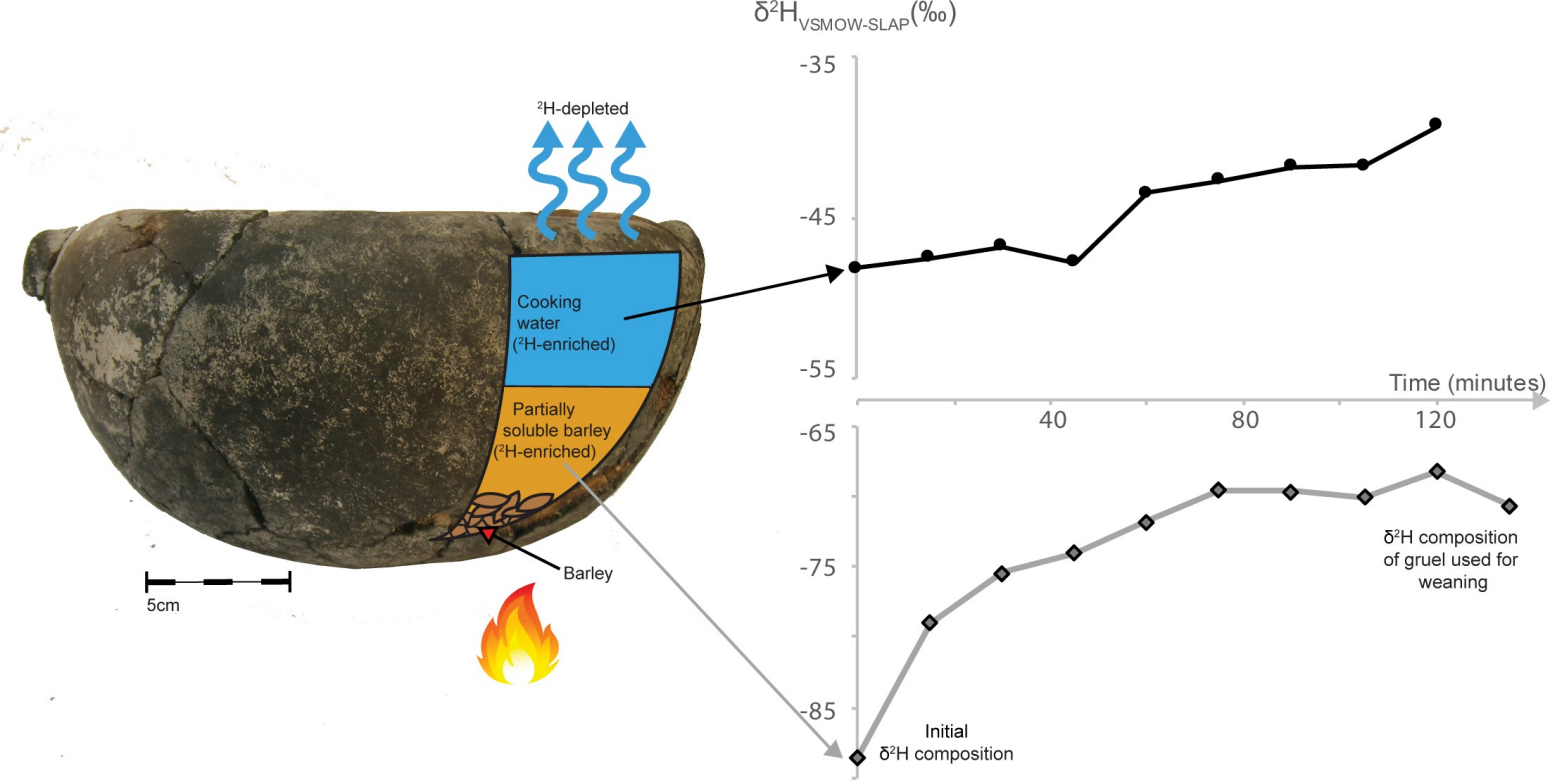
The time progression (mins) of hydrogen isotopes of cooking water and partially-soluble barley fractions of an experimental barley-water gruel. Both the cooking water and the partially-soluble barley fraction become elevated in ^2^H as a result of cooking. Data points are straight-line joined. The hydrogen isotopes of the insoluble barley fraction (not shown) are not progressively fractionated over the cooking duration (See S6 Table for data).

The δ^2^H values of the partially-soluble fraction of barley, i.e. the food fraction that would likely have been consumed by an infant being weaned, and the cooking water **(**Fig 4) become increasingly elevated in ^2^H over the cooking time (S6 Table). Barley gruel is therefore a potential source of high δ^2^H dietary inputs which could subsequently be reflected in tooth dentin. A major factor that controls the starting point and duration of infant weaning is the availability of a suitable supplementary food that has an appropriate nutrient profile and is easy to swallow and digest. The type of food that was used for weaning in Punic Sardinia could very likely have been a grain-based porridge. However, it is likely that any grain treated in the above manner would result in an increase in δ^2^H values in both the water and partially solubilized grain.

Brewing, boiling, and stewing have been shown to result in an increase in the δ^18^O of stew water of 10.3‰ on average [46]. Tuross et al., (2017) [47] document the change in the δ^18^O of biochemical constituents in foods (beef and sweet potatoes) through application of heat, and showed a change in δ^18^O of Δ 6‰ pre- and post-cooking in the most extreme case. Here, we confirm that the fractionation of hydrogen isotopes occurs as a result of cooking both in the water, as expected [73] and in the partially solubilized grain. Accordingly, one potential cause of elevated hydrogen isotope values in dentin is the consumption of heavily cooked barley gruel.

## Conclusions

The duration of the breastfeeding and weaning period varies widely depending on cultural, biological and environmental factors [74,75]. This study provides a new powerful method for examining the decline in the consumption of mother’s breastmilk and the introduction of weaning foods. Reconstructing breastfeeding and weaning practices of ancient populations can be accomplished by sequential isotope measurement of incremental tooth dentin from adult M1 permanent molars, obviating the need for infant skeletal material. The fine-grained pattern of increasing δ^2^H and declining δ^15^N and δ^13^C in dentin documents the gradual introduction of weaning foods over a duration of one to two years in the Villamar individuals.

Dentin laid down in early infancy has a lower δ^2^H value consistent with breastmilk intake. In the following dentin sections δ^2^H rises sharply with the concurrent decline in δ^15^N and δ^13^C values. All three isotopic ratios are consistent with a substitute weaning food dominated by plant source(s), and we provide evidence for the source of the increase in δ^2^H during the later first, second and third year of life as consistent with a food such as cooked grains, and inconsistent with substantial animal products. Given the large amplitude shifts observed in δ^2^H values in sequential dentin samples, consistent with likely dietary inputs (breastmilk and cooked barley porridge), this isotopic tracer reveals more detailed and potentially new and otherwise untraceable dietary sources in infancy and early childhood.

## Supporting information

S1 Appendix Villamar site details. (PDF)

S1 Table List of individuals with key information pertaining to the sample analyses from each. (DOCX)

S2 Table Radiocarbon dates of selected Villamar individuals. (DOCX)

S3 Table Hydrogen, nitrogen and carbon isotope values, standard deviations and sample numbers of individual dentin sections taken from the first permanent molars of six individuals from Villamar. *δ^2^H analysed at Harvard were converted to equivalent Cr-reactor run values using a conversion factor presented by Reynard et al., 2019[67].
(DOCX)

S4 Table Bone collagen hydrogen, nitrogen and carbon isotope values of Villamar humans and caprids. (DOCX)

S5 Table Hydrogen isotope values (‰) of infant feces, maternal breastmilk and urine (chromium (Cr)-packed reactor), and local meteoric water which were collected over a period of ~5 months. (DOCX)

S6 Table Hydrogen isotope values (‰) of barley cooking experiment fractions. *Glassy-C reactor configuration.
(DOCX)
