# Supplementary material for: Growing up in Ancient Sardinia: Infant-toddler dietary changes revealed by the novel use of hydrogen isotopes (δ2H)
Source: PLoS One. 2020 Jul 8;15(7):e0235080. doi: 10.1371/journal.pone.0235080 (PMC7343138; doi:10.1371/journal.pone.0235080)
Supplement: S1 Appendix — (PDF) [file pone.0235080.s001.pdf]

S1 Appendix. Villamar site details

The archaeological remains under study were excavated from an ancient cemetery dating to the Punic period, located in the modern town of Villamar in Sardinia, Italy. Extending research carried out in the 1990's [1], recent and ongoing excavations have focussed on 25 tombs, with the full extent of the cemetery still unknown [2,3]. Many of these funerary structures that were used for burial (chamber tombs or rock-cut niches) were later re-used to accommodate cinerary urns. In some cases, the deceased were placed in ovoid or square trench graves, while, unusually for the Punic period, there were also a number of '*alla cappuccina*' burials, which were covered by roof tiles. Children were mostly buried in amphorae, or *enchytrismos* burial [4]. Equally unusual is the deposition of children, in particular new-born babies, together with adults in two of the chamber tombs (T.16 and T.12). All the individuals sampled come from the same rock-cut chamber with a shaft entryway, tomb T.16, which was in use between the 4th and early 2nd century BCE [2,3,5]. They represent four adults and two juveniles, whose permanent first molars were collected for study together with a bone section (see S1 Table for details).

References

1. Paderi, M. C. & A. Siddu GU. Ricerche nell'abitato di Mara. Notizia preliminare sull'area della necropoli di San Pietro. In: Murgia G, editor. Villamar Una comunità, la sua storia, 121–157. Grafica del Parteolla, Dolianova; 1993.
2. Pompianu E. Nuovi scavi nella necropoli punica di Villamar (2013-2015). FOLD&R it. 2017;395: 1–28. Available: http://www.fastionline.org
3. Pompianu E, Murgia C. Nuovi scavi nella necropoli punica di Villamar. Un primo bilancio delle ricerche 2013-2015. In Serreli G., Melis R.T., French C. Sulas F. (eds.), Sa massarì. Ecologia storica dei sistemi di lavoro contadino in Sardegna («Europa e Mediterraneo, Storia e immagini di una comunità internazionale», 37), Cagliari; 2017. pp.455-504.
4. Guirguis M, Pla Orquín R, Pompianu E. Premature deaths in Punic Sardinia. Perception of childhood in funerary contexts from Monte Sirai and Villamar. In: Tabolli J, editor. From Invisible to Visible New data and Methods for the Archaeology of infant and Child Burials in Pre-Roman Italy and Beyond. Studies in Mediterranean Archaeology 149, Astrom Editions, Nicosia; 2018. pp. 207–215.
5. Pompianu E. Cartagine in Sardegna. Nota su alcuni contesti con incinerazioni dalla necropoli di Villamar. Folia Phoenicia. 2019;3: 99–116.
