# Supplementary material for: Growing up in Ancient Sardinia: Infant-toddler dietary changes revealed by the novel use of hydrogen isotopes (δ2H)
Source: PLoS One. 2020 Jul 8;15(7):e0235080. doi: 10.1371/journal.pone.0235080 (PMC7343138; doi:10.1371/journal.pone.0235080)
Supplement: S1 Table — (DOCX) [file pone.0235080.s002.docx]

**S1 Table. List of individuals with key information pertaining to the sample analyses from each.**

| Tomb | Age bracket | Burial No. | Individual No. | Bone Type | Tooth | Max/Mand | Sin/Dex |
| --- | --- | --- | --- | --- | --- | --- | --- |
| 16 | Adult | US 324 | Isloated INV #209 | Petrous | Permanent M1 | Max | Dex |
| 16 | Adult | US 327 | CR 4 | Petrous | Permanent M1 | Max | Sin |
| 16 | Adult | US 327 | CR 3 | Petrous | Permanent M1 | Max | Dex |
| 16 | Adult | US 323 | CR 1 | Femur | Permanent M1 | Mand | Dex |
| 16 | Juvenile | US 320 ID1 | CR 1 | Mandible | Permanent M1 | Mand | Sin |
| 16 | Juvenile | US 319 | CR 2 | Mandible | Permanent M1 | Mand | Dex |
