# Supplementary material for: Growing up in Ancient Sardinia: Infant-toddler dietary changes revealed by the novel use of hydrogen isotopes (δ2H)
Source: PLoS One. 2020 Jul 8;15(7):e0235080. doi: 10.1371/journal.pone.0235080 (PMC7343138; doi:10.1371/journal.pone.0235080)
Supplement: S2 Table — (DOCX) [file pone.0235080.s003.docx]

**S2 Table. Radiocarbon dates of selected Villamar individuals.**

| **AA** | **Sample ID** | **Material** | **Fraction Modern** | **14C age BP** | **Calendar Age Range (95%)** |
| --- | --- | --- | --- | --- | --- |
| AA113254 | V323CR1 | collagen | 0.7509 +- 0.0020 | 2301 +- 21 | 2354-2212 cal BP |
| AA113255 | V319CR2 | collagen | 0.7488 +- 0.0019 | 2323 +- 21 | 2357-2324 cal BP |
| AA113256 | V323CR1B | collagen | 0.7535 +- 0.0022 | 2274 +- 23 | 2349-2180 cal BP |
| AA113257 | V324CR3 | collagen | 0.7550 +- 0.0019 | 2258 +- 20 | 2344-2160 cal BP |
| AA113258 | V322CR5 | collagen | 0.7483 +- 0.0020 | 2329 +- 21 | 2359-2327 cal BP |
| AA113259 | V324CR2 | collagen | 0.7578 +- 0.0023 | 2228 +- 24 | 2365-1997 cal BP |
| AA113260 | V327CR3 | collagen | 0.7537 +- 0.0034 | 2271 +- 36 | 2350-2157 cal BP |
