# Supplementary material for: Growing up in Ancient Sardinia: Infant-toddler dietary changes revealed by the novel use of hydrogen isotopes (δ2H)
Source: PLoS One. 2020 Jul 8;15(7):e0235080. doi: 10.1371/journal.pone.0235080 (PMC7343138; doi:10.1371/journal.pone.0235080)
Supplement: S3 Table — *δ2H analysed at Harvard were converted to equivalent Cr-reactor run values using a conversion factor presented by Reynard et al., 2019[67]. (DOCX) [file pone.0235080.s004.docx]

**S3 Table. Hydrogen, nitrogen and carbon isotope values, standard deviations and sample numbers of individual dentin sections taken from the first permanent molars of six individuals from Villamar.** *δ^2^H analysed at Harvard were converted to equivalent Cr-reactor run values using a conversion factor presented by Reynard et al., 2019[67]

| **Sample ID** | **Lab** | **Dentin section** | **δ^2^H Cr-reactor** | **δ^2^H glassy-C** | **δ^2^H SD** | **No. of analyses (δ^2^H)** | **δ^15^N** | **δ^15^N SD** | **Amt%N** | **mean δ^13^C VPDB** | **δ^13^C SD** | **Amt%C** | **Atom C/N** | **No. of analyses (C&N)** |
| --- | --- | --- | --- | --- | --- | --- | --- | --- | --- | --- | --- | --- | --- | --- |
| 327 CR3 | Harvard* | 1 | 1 | -9 |  | 1 |  |  |  |  |  |  |  |  |
|  |  | 2 | 19 | 9 | 2 | 2 | 15.5 |  | 15.6 | -18.8 |  | 42.8 | 3.2 | 1 |
|  |  | 3 | 18 | 8 | 3 | 2 | 13.0 |  | 15.3 | -19.2 |  | 42.4 | 3.2 | 1 |
|  |  | 4 | 14 | 4 |  | 1 | 13.2 |  | 15.6 | -19.3 |  | 43.1 | 3.2 | 1 |
|  |  | 5 | 16 | 6 | 1 | 2 | 12.5 | 0.1 | 15.8 | -19.7 | 0.0 | 43.8 | 3.2 | 2 |
|  |  | 6 | 18 | 8 |  | 1 | 12.3 | 0.2 | 15.8 | -19.9 | 0.1 | 43.6 | 3.2 | 2 |
|  |  | 7 | 17 | 7 |  | 1 | 12.6 |  | 15.4 | -19.6 |  | 42.4 | 3.2 | 1 |
|  |  | 8 | 14 | 4 |  | 1 | 12.9 |  | 15.5 | -19.7 |  | 43.2 | 3.2 | 1 |
|  |  | 9 | 5 | -5 |  | 1 | 12.8 |  | 15.6 | -19.9 |  | 43.1 | 3.2 | 1 |
|  |  | 10 | 3 | -7 |  | 1 | 12.1 |  | 15.5 | -20.0 |  | 42.9 | 3.2 | 1 |
|  |  | 11 | 2 | -8 |  | 1 | 12.5 |  | 15.3 | -20.0 |  | 42.3 | 3.2 | 1 |
|  |  | 12 | 2 | -8 |  | 1 | 12.3 |  | 15.4 | -20.0 |  | 42.6 | 3.2 | 1 |
|  |  | 13 | 3 | -7 |  | 1 | 12.6 |  | 15.4 | -20.0 |  | 42.6 | 3.2 | 1 |
|  |  | 14 | 5 | -5 |  | 1 | 12.4 |  | 15.5 | -19.9 |  | 42.8 | 3.2 | 1 |
|  |  | 15 | 5 | -5 |  | 1 | 12.4 |  | 15.4 | -19.6 |  | 42.8 | 3.2 | 1 |
|  |  | 16 | 1 | -9 |  | 1 | 12.1 |  | 15.5 | -19.4 |  | 42.6 | 3.2 | 1 |
|  |  | 17 | 3 | -7 |  | 1 | 12.0 |  | 15.5 | -19.6 |  | 42.7 | 3.2 | 1 |
|  |  | 18 | 5 | -5 | 1 | 2 | 12.4 |  | 15.2 | -19.5 |  | 42.0 | 3.2 | 1 |
|  |  | 19 | 7 | -3 |  | 1 | 12.9 |  | 15.4 | -19.6 |  | 42.3 | 3.2 | 1 |
|  |  | 20 | 3 | -7 |  | 1 | 12.7 |  | 15.5 | -19.6 |  | 42.5 | 3.2 | 1 |
|  |  | 21 | 8 | -2 |  | 1 | 12.8 |  | 15.2 | -19.4 |  | 41.7 | 3.2 | 1 |
|  |  | 22 |  |  |  |  |  |  |  |  |  |  |  |  |
| 319 CR2 | Harvard* | 1 |  |  |  |  | 15.7 |  | 16.6 | -16.4 |  | 45.4 | 3.2 | 1 |
|  |  | 2 | 1 | -9 |  | 1 | 14.8 |  | 16.7 | -17.3 |  | 46.1 | 3.2 | 1 |
|  |  | 3 | 11 | 1 | 2 | 2 | 13.0 |  | 16.8 | -17.9 |  | 46.1 | 3.2 | 1 |
|  |  | 4 | 14 | 4 | 1 | 2 | 12.3 | 0.1 | 16.9 | -18.2 | 0.0 | 46.5 | 3.2 | 2 |
|  |  | 5 | 14 | 4 |  | 1 | 12.3 | 0.1 | 16.8 | -18.4 | 0.1 | 46.1 | 3.2 | 2 |
|  |  | 6 | 12 | 2 |  | 1 | 12.3 | 0.1 | 17.1 | -18.7 | 0.0 | 46.7 | 3.2 | 2 |
|  |  | 7 | 18 | 8 |  | 1 | 12.6 | 0.1 | 16.8 | -18.8 | 0.0 | 46.3 | 3.2 | 2 |
|  |  | 8 | 19 | 9 |  |  | 12.0 | 0.2 | 16.7 | -19.3 | 0.0 | 46.1 | 3.2 | 2 |
|  |  | 9 | 10 | 0 |  |  | 11.9 |  | 16.5 | -19.1 |  | 45.7 | 3.2 | 1 |
|  |  | 10 |  |  |  |  | 11.9 |  | 16.0 | -19.0 |  | 44.3 | 3.2 | 1 |
|  |  | 11 |  |  |  |  | 12.0 |  | 16.3 | -19.0 |  | 45.4 | 3.2 | 1 |
|  |  | 12 |  |  |  |  | 12.3 |  | 16.3 | -18.8 |  | 45.3 | 3.2 | 1 |
|  |  | 13 |  |  |  |  |  |  |  |  |  |  |  |  |
| 323 CR1 | Harvard* | 1 |  |  |  |  | 16.3 |  | 16.8 | -17.3 |  | 45.2 | 3.1 | 1 |
|  |  | 2 | -8 | -18 | 4 | 2 | 16.1 | 0.2 | 16.6 | -17.8 | 0.1 | 44.7 | 3.1 | 2 |
|  |  | 3 | -4 | -14 | 6 | 2 | 15.4 | 0.5 | 16.6 | -18.1 | 0.1 | 44.9 | 3.2 | 2 |
|  |  | 4 | 0 | -10 |  | 1 | 14.7 |  | 16.6 | -18.3 |  | 44.6 | 3.1 | 1 |
|  |  | 5 | 0 | -10 |  | 1 | 13.8 | 0.2 | 16.8 | -18.6 | 0.0 | 45.6 | 3.2 | 2 |
|  |  | 6 | 9 | -1 |  | 1 | 13.2 | 0.1 | 16.4 | -18.8 | 0.0 | 44.3 | 3.2 | 2 |
|  |  | 7 | 15 | 5 |  | 1 | 12.7 | 0.1 | 16.6 | -18.8 | 0.1 | 44.8 | 3.2 | 2 |
|  |  | 8 | 16 | 6 |  | 1 | 12.2 | 0.1 | 16.6 | -18.8 | 0.0 | 44.9 | 3.2 | 2 |
|  |  | 9 | 13 | 3 |  | 1 | 12.2 | 0.0 | 16.4 | -18.7 | 0.1 | 44.4 | 3.2 | 2 |
|  |  | 10 | 8 | -2 |  | 1 | 12.2 | 0.1 | 16.7 | -18.8 | 0.1 | 45.4 | 3.2 | 2 |
|  |  | 11 | 10 | 0 | 2 | 2 | 12.4 |  | 16.5 | -18.6 |  | 45.0 | 3.2 | 1 |
|  |  | 12 | 9 | -1 |  | 1 | 12.3 | 0.1 | 16.4 | -18.7 | 0.0 | 44.6 | 3.2 | 2 |
|  |  | 13 | 4 | -6 |  | 1 | 12.4 |  | 16.7 | -18.7 |  | 45.4 | 3.2 | 1 |
|  |  | 14 | 1 | -9 |  | 1 | 12.2 |  | 16.3 | -18.6 |  | 44.6 | 3.2 | 1 |
|  |  | 15 | 0 | -10 |  | 1 | 11.8 |  | 16.6 | -18.4 |  | 45.3 | 3.2 | 1 |
|  |  | 16 | -1 | -11 |  | 1 | 11.3 |  | 16.5 | -18.4 |  | 45.3 | 3.2 | 1 |
|  |  | 17 |  |  |  |  | 11.5 |  | 16.5 | -18.6 |  | 45.3 | 3.2 | 1 |
|  |  | 18 |  |  |  |  | 11.4 |  | 16.2 | -18.9 |  | 44.4 | 3.2 | 1 |
|  |  | 19 |  |  |  |  | 11.5 |  | 16.1 | -19.3 |  | 44.1 | 3.2 | 1 |
|  |  | 20 |  |  |  |  |  |  |  |  |  |  |  |  |
| 320 1D1 | USGS | 1 | -22 |  |  |  | 16.4 |  | 15.5 | -17.5 |  | 42.7 | 3.2 | 1 |
|  |  | 2 | -10 |  |  |  | 15.2 |  | 15.8 | -17.6 |  | 43.7 | 3.2 | 1 |
|  |  | 3 | 3 |  |  |  | 13.9 | 1.3 | 15.6 | -17.5 | 0.1 | 43.0 | 3.2 | 2.0 |
|  |  | 4 | 4 |  |  |  | 13.0 |  | 15.6 | -17.5 |  | 42.9 | 3.2 | 1 |
|  |  | 5 | 6 |  |  |  | 13.0 |  | 15.7 | -17.5 |  | 43.2 | 3.2 | 1 |
|  |  | 6 | 8 |  |  |  | 12.2 |  | 15.8 | -17.7 |  | 42.9 | 3.2 | 1 |
|  |  | 7 | 8 |  |  |  | 11.9 | 0.1 | 15.5 | -17.8 | 0.0 | 42.3 | 3.2 | 2.0 |
|  |  | 8 | 8 |  |  |  | 11.3 |  | 15.5 | -18.0 |  | 42.4 | 3.2 | 1 |
|  |  | 9 | -3 |  |  |  | 11.3 |  | 15.4 | -18.0 |  | 42.0 | 3.2 | 1 |
|  |  | 10 | -6 |  |  |  | 11.0 |  | 15.3 | -17.8 |  | 42.0 | 3.2 | 1 |
|  |  | 11 | -9 |  |  |  | 10.9 |  | 15.2 | -17.9 |  | 41.9 | 3.2 | 1 |
|  |  | 12 | -10 |  |  |  | 10.7 |  | 15.4 | -17.6 |  | 42.3 | 3.2 | 1 |
|  |  | 13 | -13 |  |  |  | 10.8 |  | 15.3 | -18.0 |  | 41.9 | 3.2 | 1 |
|  |  | 14 | -13 |  |  |  | 10.3 |  | 15.1 | -17.8 |  | 40.9 | 3.2 | 1 |
|  |  | 15 | -12 |  |  |  | 11.1 |  | 15.4 | -17.9 |  | 41.9 | 3.2 | 1 |
| 327 CR4 | USGS | 1 | -2 |  |  |  | 14.6 |  | 14.8 | -18.0 |  | 41.1 | 3.2 | 1 |
|  |  | 2 | 0 |  |  |  | 13.7 |  | 15.3 | -18.2 |  | 42.3 | 3.2 | 1 |
|  |  | 3 | 4 |  |  |  | 12.5 |  | 15.7 | -18.6 |  | 43.3 | 3.2 | 1 |
|  |  | 4 | 6 |  |  |  | 11.1 | 0.3 | 14.7 | -18.7 | 0.0 | 40.5 | 3.2 | 2 |
|  |  | 5 | 8 |  |  |  | 10.7 |  | 14.7 | -18.7 |  | 40.3 | 3.2 | 1 |
|  |  | 6 | 9 |  |  |  | 10.7 | 0.1 | 15.3 | -18.9 | 0.0 | 41.8 | 3.2 | 2 |
|  |  | 7 | 10 |  |  |  | 10.8 |  | 15.5 | -19.0 |  | 42.5 | 3.2 | 1 |
|  |  | 8 | 5 |  |  |  | 10.5 |  | 15.4 | -18.9 |  | 42.3 | 3.2 | 1 |
|  |  | 9 | 5 |  |  |  | 10.4 |  | 15.6 | -18.9 |  | 42.9 | 3.2 | 1 |
|  |  | 10 | 12 |  |  |  | 10.4 |  | 15.1 | -18.5 |  | 42.0 | 3.2 | 1 |
|  |  | 11 | 11 |  |  |  | 10.6 |  | 15.1 | -18.4 |  | 41.7 | 3.2 | 1 |
|  |  | 12 | 7 |  |  |  | 10.9 |  | 15.2 | -18.7 |  | 42.2 | 3.2 | 1 |
|  |  | 13 |  |  |  |  |  |  |  |  |  |  |  |  |
|  |  | 14 |  |  |  |  |  |  |  |  |  |  |  |  |
|  |  | 15 |  |  |  |  |  |  |  |  |  |  |  |  |
|  |  | 16 |  |  |  |  |  |  |  |  |  |  |  |  |
|  |  | 17 |  |  |  |  |  |  |  |  |  |  |  |  |
| 324 INV 209 | USGS | 1 | -4 |  |  |  | 15.2 |  | 15.1 | -17.7 |  | 41.1 | 3.2 | 1 |
|  |  | 2 | -2 |  |  |  | 15.6 |  | 15.6 | -17.8 |  | 42.3 | 3.2 | 1 |
|  |  | 3 | 8 |  |  |  | 14.3 | 0.8 | 15.2 | -18.5 | 0.2 | 41.4 | 3.2 | 2 |
|  |  | 4 | 7 |  |  |  | 13.2 |  | 15.6 | -19.2 |  | 42.5 | 3.2 | 1 |
|  |  | 5 | 10 |  |  |  | 13.3 | 0.3 | 15.3 | -19.3 | 0.1 | 41.7 | 3.2 | 2 |
|  |  | 6 | 12 |  |  |  | 12.7 |  | 15.2 | -19.4 |  | 41.4 | 3.2 | 1 |
|  |  | 7 | 13 |  |  |  | 12.0 |  | 15.2 | -19.2 |  | 41.5 | 3.2 | 1 |
|  |  | 8 | 11 |  |  |  | 11.9 |  | 15.3 | -19.2 |  | 41.8 | 3.2 | 1 |
|  |  | 9 | 7 |  |  |  | 11.5 |  | 15.1 | -19.0 |  | 41.3 | 3.2 | 1 |
|  |  | 10 | 4 |  |  |  | 11.5 |  | 15.1 | -19.1 |  | 41.2 | 3.2 | 1 |
|  |  | 11 | 1 |  |  |  | 11.0 |  | 14.5 | -18.7 |  | 40.0 | 3.2 | 1 |
|  |  | 12 | 1 |  |  |  | 11.5 |  | 14.5 | -18.9 |  | 40.1 | 3.2 | 1 |
|  |  | 13 | -2 |  |  |  | 11.9 |  | 14.8 | -18.8 |  | 40.9 | 3.2 | 1 |
|  |  | 14 | -1 |  |  |  | 11.9 |  | 14.5 | -18.8 |  | 39.9 | 3.2 | 1 |
|  |  | 15 | -1 |  |  |  | 11.8 |  | 14.6 | -19.0 |  | 40.5 | 3.2 | 1 |
|  |  | 16 |  |  |  |  |  |  |  |  |  |  |  |  |
|  |  | 17 |  |  |  |  |  |  |  |  |  |  |  |  |
|  |  | 18 |  |  |  |  |  |  |  |  |  |  |  |  |
