# Supplementary material for: Growing up in Ancient Sardinia: Infant-toddler dietary changes revealed by the novel use of hydrogen isotopes (δ2H)
Source: PLoS One. 2020 Jul 8;15(7):e0235080. doi: 10.1371/journal.pone.0235080 (PMC7343138; doi:10.1371/journal.pone.0235080)
Supplement: S4 Table — (DOCX) [file pone.0235080.s005.docx]

**S4 Table. Bone collagen hydrogen, nitrogen and carbon isotope values of Villamar humans and caprids.**

| **Sample ID** | **Cr δ^2^H** | **Cr δ^2^H SD** | **δ^15^N** | **δ^15^N SD** | **δ^13^C** | **δ^13^C SD** | **No. of analyses** |
| --- | --- | --- | --- | --- | --- | --- | --- |
| 327 CR3 | 12 | 5 | 11.8 | 0.0 | -19.1 | 0.2 | 2 |
| 319 CR2 | 3 | 2 | 11.5 | 0.0 | -19.0 | 0.6 | 2 |
| 323 CR1 | 8 | 2 | 12.0 | 0.2 | -19.0 | 0.2 | 2 |
| 320 ID1 | 8 | 4 | 11.4 | 0.1 | -18.3 | 0.2 | 2 |
| 327 CR4 | 14 | 1 | 11.7 | 0.1 | -18.1 | 0.1 | 2 |
| 324 INV 209 | -2 | 2 | 10.7 | 0.0 | -19.1 | 0.1 | 2 |
| 319 CAP A | -37 | 1 | 11.6 | 0.1 | -20.5 | 0.6 | 2 |
| 319 CAP B | -37 | 0 | 9.8 | 0.1 | -20.7 | 0.2 | 2 |
| 319 CAP C | -37 | 2 | 10.2 | 0.4 | -20.7 | 0.3 | 2 |
