# Supplementary material for: Growing up in Ancient Sardinia: Infant-toddler dietary changes revealed by the novel use of hydrogen isotopes (δ2H)
Source: PLoS One. 2020 Jul 8;15(7):e0235080. doi: 10.1371/journal.pone.0235080 (PMC7343138; doi:10.1371/journal.pone.0235080)
Supplement: S5 Table — (DOCX) [file pone.0235080.s006.docx]

**S5 Table. Hydrogen isotope values (‰) of infant feces, maternal breastmilk and urine (chromium (Cr)-packed reactor), and local meteoric water which were collected over a period of ~5 months.**

| **Order of**  **collection** | **δ^2^H ‰**  **Infant feces** | **δ^2^H ‰**  **BM lipid** | **δ^2^H ‰**  **BM stock mixed** | **δ^2^H ‰**  **BM stock lipid-depleted** | | **δ^2^H ‰**  **BM water** | **δ^2^H ‰**  **Maternal urine water** | **δ^2^H ‰**  **Maternal urine solid** | | **δ^2^H ‰**  **Local meteoric water** |
| --- | --- | --- | --- | --- | --- | --- | --- | --- | --- | --- |
| 1 |  |  |  |  |  | |  | |  | -42 |
| 2 | -193 | -206 | -160 | -63 |  | |  | |  |  |
| 3 |  |  |  |  | -36 | | -34 | | -1 |  |
| 4 |  |  |  |  |  | | -36 | | 71 |  |
| 5 | -123 | -203 | -111 | -68 | -37 | | -39 | | 92 |  |
| 6 | -138 | -204 | -106 | -119 | -38 | | -38 | | 28 |  |
| 7 |  |  |  |  |  | | -37 | | 50 | -42 |
| 8 | -144 | -157 | -117 | -91 |  | | -34 | | 35 |  |
| 9 |  |  |  |  |  | | -34 | | 41 |  |
| 10 | -127 | -104 | -73 | -59 | -37 | |  | |  |  |
| **Average:** | **-145** | **-175** | **-114** | **-80** | **-37** | | **-36** | | **45** | **-42** |
| **SD:** | **28** | **44** | **31** | **25** | **1** | | **2** | | **30** | **1** |
