# Supplementary material for: Growing up in Ancient Sardinia: Infant-toddler dietary changes revealed by the novel use of hydrogen isotopes (δ2H)
Source: PLoS One. 2020 Jul 8;15(7):e0235080. doi: 10.1371/journal.pone.0235080 (PMC7343138; doi:10.1371/journal.pone.0235080)
Supplement: S6 Table — *Glassy-C reactor configuration. (DOCX) [file pone.0235080.s007.docx]

**S6 Table. Hydrogen isotope values (‰) of barley cooking experiment fractions.** *Glassy-C reactor configuration

| Time (mins) | δ^2^H ‰  Cooking water | δ^2^H ‰  Partially-soluble barley* | δ^2^H ‰  Insoluble barley* | |
| --- | --- | --- | --- | --- |
| 0 | -48 | -84 | | -65 |
| 15 | -48 | -74 | | -72 |
| 30 | -47 | -70 | | -74 |
| 45 | -48 | -69 | | -67 |
| 60 | -43 | -67 | | -68 |
| 75 | -43 | -65 | | -70 |
| 90 | -42 | -65 | | -73 |
| 105 | -42 | -65 | | -68 |
| 120 | -39 | -63 | | -67 |
| 135 |  | -66 | | -69 |
